# A Novel Quorum-Quenching *N*-Acylhomoserine Lactone Acylase from Acidovorax sp. Strain MR-S7 Mediates Antibiotic Resistance

**DOI:** 10.1128/AEM.00080-17

**Published:** 2017-06-16

**Authors:** Hiroyuki Kusada, Hideyuki Tamaki, Yoichi Kamagata, Satoshi Hanada, Nobutada Kimura

**Affiliations:** a Bioproduction Research Institute, National Institute of Advanced Industrial Science and Technology (AIST), Tsukuba, Ibaraki, Japan; b Graduate School of Life and Environmental Sciences, University of Tsukuba, Tsukuba, Ibaraki, Japan; c JST ERATO Nomura Microbial Community Control Project, University of Tsukuba, Tsukuba, Ibaraki, Japan; University of Tokyo

**Keywords:** β-lactam antibiotics, β-lactam acylase, *N*-acylhomoserine lactones, quorum quenching, quorum sensing

## Abstract

*N*-Acylhomoserine lactone acylase (AHL acylase) is a well-known enzyme responsible for disrupting cell-cell communication (quorum sensing) in bacteria. Here, we isolated and characterized a novel and unique AHL acylase (designated MacQ) from a multidrug-resistant bacterium, Acidovorax sp. strain MR-S7. The purified MacQ protein heterologously expressed in Escherichia coli degraded a wide variety of AHLs, ranging from C_6_ to C_14_ side chains with or without 3-oxo substitutions. We also observed that AHL-mediated virulence factor production in a plant pathogen, Pectobacterium carotovorum, was dramatically attenuated by coculture with MacQ-overexpressing Escherichia coli, whereas E. coli with an empty vector was unable to quench the pathogenicity, which strongly indicates that MacQ can act *in vivo* as a quorum-quenching enzyme and interfere with the quorum-sensing system in the pathogen. In addition, this enzyme was found to be capable of degrading a wide spectrum of β-lactams (penicillin G, ampicillin, amoxicillin, carbenicillin, cephalexin, and cefadroxil) by deacylation, clearly indicating that MacQ is a bifunctional enzyme that confers both quorum quenching and antibiotic resistance on strain MR-S7. MacQ has relatively low amino acid sequence identity to any of the known acylases (<39%) and has among the broadest substrate range. Our findings provide the possibility that AHL acylase genes can be an alternative source of antibiotic resistance genes posing a threat to human health if they migrate and transfer to pathogenic bacteria.

**IMPORTANCE** *N*-Acylhomoserine lactones (AHLs) are well-known signal molecules for bacterial cell-cell communication (quorum sensing), and AHL acylase, which is able to degrade AHLs, has been recognized as a major target for quorum-sensing interference (quorum quenching) in pathogens. In this work, we succeeded in isolating a novel AHL acylase (MacQ) from a multidrug-resistant bacterium and demonstrated that the MacQ enzyme could confer multidrug resistance as well as quorum quenching on the host organism. Indeed, the purified MacQ protein was found to be bifunctional and capable of degrading not only various AHL derivatives but also multiple β-lactam antibiotics by deacylation activities. Although quorum quenching and antibiotic resistance have been recognized to be distinct biological functions, our findings clearly link the two functions by discovering the novel bifunctional enzyme and further providing the possibility that a hitherto-overlooked antibiotic resistance mechanism mediated by the quorum-quenching enzyme may exist in natural environments and perhaps in clinical settings.

## INTRODUCTION

A diverse array of microorganisms communicate with each other in a cell density-dependent manner through the exchange of diffusible signal molecules called autoinducers, e.g., *N*-acylhomoserine lactones (AHLs), in some Gram-negative bacteria and in small peptides in Gram-positive bacteria, respectively (1). This intercellular communication system, termed quorum sensing (QS), is responsible for the regulation of gene expression involved in pathogenicity (2), biofilm formation (3), secondary metabolite production (4), motility (5), and respiration (6). In particular, since a number of different pathogens utilize AHLs to control the production of virulence factors and biofilm formation, which are serious problems in nosocomial infectious diseases (7, 8), it is expected that disruption of the QS system (i.e., quorum quenching) could inhibit bacterial infectious diseases (9).

**FIG 1:**
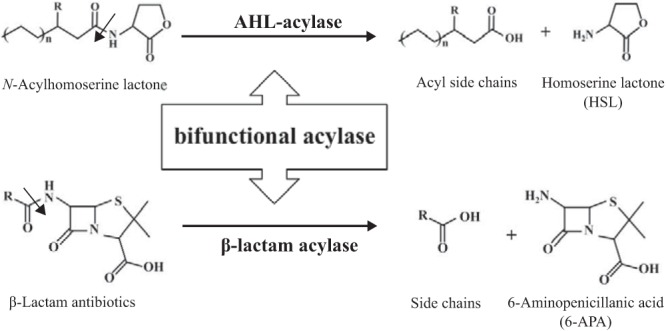
Schematic representation of *N*-acylhomoserine lactones (AHLs) and β-lactam antibiotic deacylation. The basic structure of AHLs and β-lactam antibiotics, and the corresponding degradation reaction mechanisms of AHL acylase (top) and β-lactam acylase (bottom) are shown.

Quorum quenching is mainly achieved through enzymatic degradation of AHLs by two different families of enzymes, AHL lactonases and AHL acylases (10). AHL acylases are well-known members of the Ntn hydrolase family proteins (11), and they inactivate AHLs by cleaving the acyl side chains from the homoserine lactone (Fig. 1). AHL acylase activity has been identified in various bacteria, including Gram-negative and Gram-positive bacteria (12–18), but it differs in substrate specificity based on the different acyl chain substitutions of AHLs.

Based on the fact that AHLs and β-lactam antibiotics are structurally similar and both have an acyl side chain, we postulate that there should be bifunctional AHL acylases that mediate β-lactam antibiotic resistance and disrupt cell-cell communications (Fig. 1); if so, hitherto-unrecognized antibiotic-resistant candidates may be present in nature. However, very little is known about bifunctional acylases and their biological functions. So far, it has been reported that only two experimentally characterized AHL acylases (AhlM from Streptomyces sp. strain M664 and *Kc*PGA from Kluyvera citrophila DMSZ 2660) catalyze penicillin G as just one of the substrates (19, 20), and their *in vivo* contributions to antibiotic resistance have never been discussed. Furthermore, the phylogenetic diversity of bifunctional acylases remains largely unclear, because the bifunctionality is rarely found.

In the present study, we found a new candidate gene encoding bifunctional acylase from a multidrug-resistant bacterium, Acidovorax sp. strain MR-S7, which is capable of inactivating AHLs (21), by cloning and expressing the gene from the bacterium and characterizing its catalytic functions and phylogenetic relatedness with previously identified acylases.

## RESULTS AND DISCUSSION

### Cloning of a putative bifunctional acylase gene and heterologous expression in E. coli.

**FIG 2:**
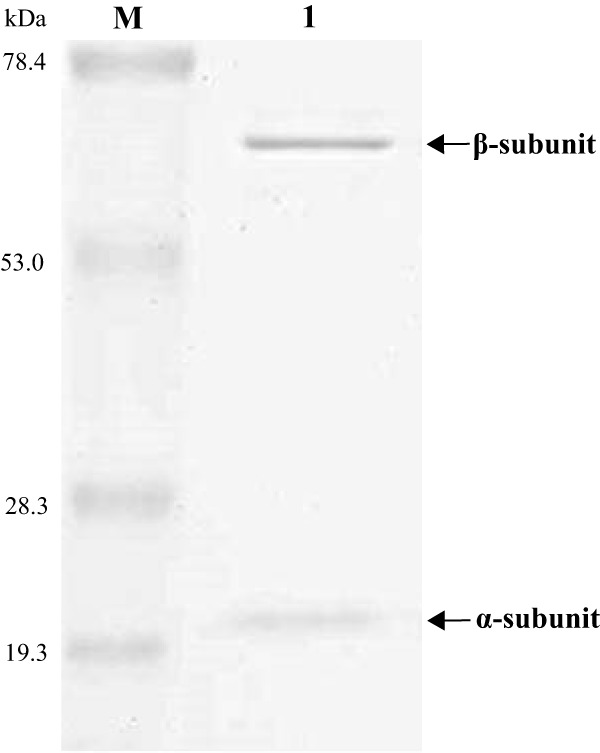
SDS-PAGE analysis of purified MacQ. The protein was purified from recombinant Escherichia coli, as described in Materials and Methods. The soluble fraction was loaded onto a 10% SDS-PAGE gel after nickel affinity chromatography. The arrows indicate the bands corresponding to two subunits of MacQ. Lane M, molecular size marker; lane 1, purified MacQ.

In the present study, we identified one putative acylase gene (*macQ*) in the strain MR-S7 genome based on homology and domain searches. In an amino acid sequence comparison, MacQ is related to Ntn hydrolase family proteins and shows the highest identity to AHL acylase (QqaR) (39%) from Deinococcus radiodurans R1 (16) among the verified acylases. Interestingly, MacQ also shares high similarity to putative β-lactam acylase (78%) from Acidovorax ebreus TPSY. Since the result implicates the potential bifunctionality of MacQ, we purified the enzyme from the recombinant Escherichia coli strain Origami 2(DE3) using a Ni affinity column. SDS-PAGE analysis showed that MacQ consisted of two subunits (α-subunit, 20 kDa; β-subunit, 62 kDa) (Fig. 2) corresponding to the predicted molecular mass (84 kDa) from the amino acid sequence (806 amino acids).

### Enzymatic properties of MacQ.

**FIG 3:**
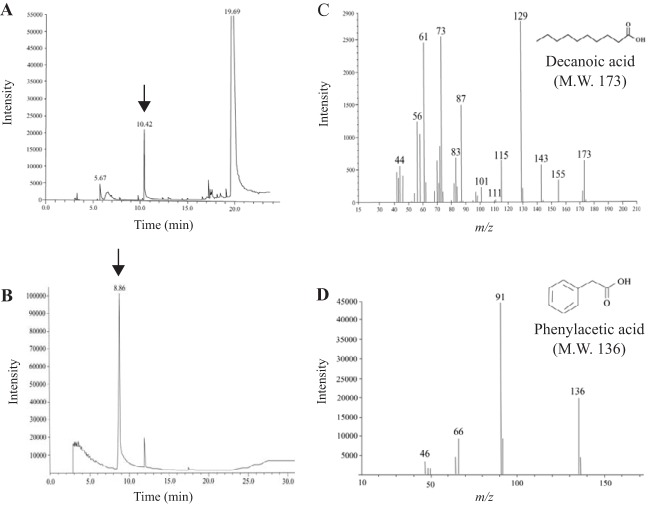
Metabolite analyses of C_10_-HSL and penicillin G degradation products by MacQ. Gas chromatography profile of digestion products of C_10_-HSL (A) and penicillin G (B) by MacQ, and their mass spectrometry retention times at 10.42 min (C) and 8.86 min (D), respectively.

To demonstrate whether MacQ can degrade both AHLs and β-lactams, gas chromatography-mass spectroscopy (GC-MS) analyses were performed to detect the presence of decanoic acid and phenylacetic acid generated by deacylation of C_10_-HSL and penicillin G, respectively. After incubation of purified MacQ with C_10_-HSL or penicillin G, we observed that each product emerged, with GC retention times of 10.42 min (Fig. 3A) and 8.86 min (Fig. 3B), respectively. MS analyses of the 10.42- and 8.86-min GC fractions showed the [M-H] ions at *m/z* 173 (Fig. 3C) and *m/z* 136 (Fig. 3D), corresponding to the molecular weights of decanoic acid and phenylacetic acid, respectively. These results obviously showed that MacQ has the bifunctional capacity to degrade both AHL and β-lactam antibiotics by deacylation activity.

**FIG 4:**
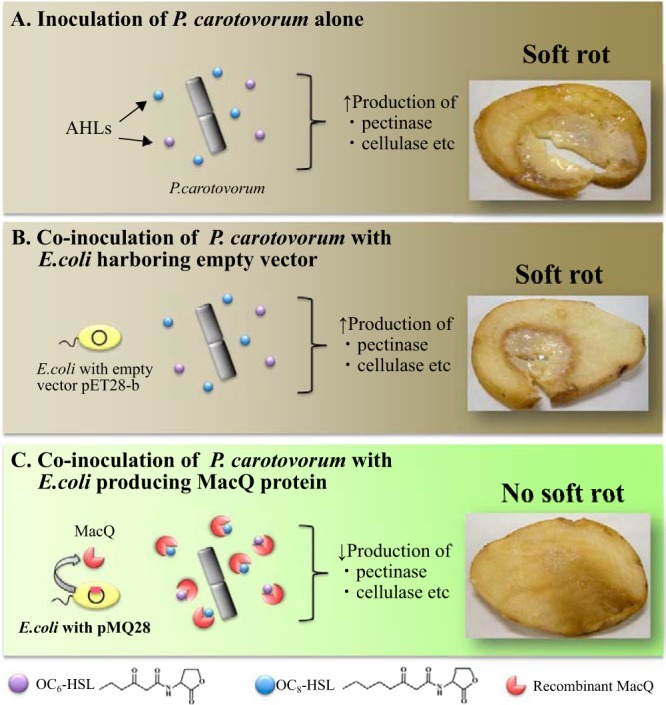
Quenching of pectinolytic activity in P. carotovorum subsp. carotovorum by MacQ. The pectinolytic activities were assessed by visual inspection of maceration zones induced by the plant pathogen P. carotovorum upon inoculation of a potato tuber. (A) Inoculation of culture fluid of P. carotovorum subsp. carotovorum alone. (B and C) Coinoculation with E. coli harboring an empty vector (B) and with E. coli expressing MacQ protein (C). P. carotovorum produces AHLs (i.e., OC_6_-HSL and OC_8_-HSL, indicated by purple and blue balls, respectively), and these AHLs positively regulate the production of plant cell wall-degrading enzymes (e.g., pectinase and cellulose) involved in development of soft rot symptoms in potato (A). (B) Since E. coli with empty vector has no direct influence on AHL-mediated plant cell wall-degrading enzyme production in P. carotovorum, soft rot symptom is observed. (C) E. coli with pMQ28, however, produces MacQ protein (red pie-shaped diagrams) capable of quenching the production of plant cell wall-degrading enzymes by degrading AHLs, and then the soft rot symptom is obviously attenuated.

We further characterized the AHL degradation capacity of purified MacQ using green fluorescent protein (GFP)-based and virulence-based AHL-detectable biosensor strains, as described in Materials and Methods. MacQ was able to inactivate all tested AHLs ranging from C_6_ to C_14_ chains with or without 3-oxo substitutions (Table 1). Besides, we observed that AHL-mediated virulence factor production in a plant pathogen, Pectobacterium carotovorum, was dramatically attenuated by coculture with MacQ-overexpressing Escherichia coli Origami 2(DE3), whereas E. coli with an empty vector was unable to quench the pathogenicity (Fig. 4). Although whether or not the production of plant cell wall-degrading enzymes was inhibited by MacQ is unclear, it is quite possible that MacQ acts as a quorum-quenching enzyme and disrupts the quorum-sensing system in the pathogen.

**TABLE 1:** Results of AHL inactivation bioassay

Protein	Microorganism	Degradation activity by AHL derivative^*a*^									Reference
		C_6_	C_8_	C_10_	C_12_	OC_6_	OC_8_	OC_10_	OC_12_	OC_14_	
MacQ	Acidovorax sp. MR-S7	+	+	+	+	+	+	+	+	+	This study
AiiD	Ralstonia sp. XJ12B	ND	ND	ND	ND	+	+	+	+	ND	12
AhlM	Streptomyces sp. M664	−	+	+	ND	−	ND	ND	+	ND	19
PvdQ	Pseudomonas aeruginosa PAO1	−	+	+	+	−	ND	ND	+	ND	17
QuiP	Pseudomonas aeruginosa PAO1	−	+	+	+	−	ND	ND	+	ND	18
HacA	Pseudomonas syringae B728a	−	+	+	+	−	ND	ND	−	ND	14
HacB	Pseudomonas syringae B728a	+	+	+	+	+	ND	ND	−	ND	14
AiiC	Anabaena sp. PCC7120	+	+	+	+	ND	ND	+	+	ND	13
Aac	Shewanella sp. MIB015	−	+	+	+	ND	ND	ND	ND	ND	15
QqaR	Deinococcus radiodurans R1	−	+	+	+	−	+	+	+	+	16
*Kc*PGA	Kluyvera citrophila DSM 2660	+	+	−	−	+	ND	ND	ND	ND	20

a *E*. coli MT102 harboring pJBA132 was used for detecting C_6_-HSL, C_8_-HSL, C_10_-HSL, C_12_-HSL, OC_6_-HSL, OC_8_-HSL, OC_10_-HSL, and OC_12_-HSL, and P. putida F117 harboring pKR-C12 was used for detecting OC_14_-HSL. The plus and minus signs indicate degradation and nondegradation activities, respectively. ND, not determined.

In addition to quorum-quenching activity, we investigated the antibiotic-resistance-conferring capability of MacQ using an MIC method. We confirmed that recombinant plasmid (pMQ28) conferred 2- to 16-fold higher MIC values toward all six β-lactam antibiotics in E. coli Origami 2(DE3) than the strain with a control vector (Table 2). This indicates that MacQ confers multiple β-lactam antibiotic resistance on a host strain by hydrolyzing β-lactam antibiotics.

**TABLE 2:** β-Lactam antibiotic resistance assay^*a*^

E. coli strain	MIC (μg/ml)					
	PENG	AMP	AMO	CAR	CEF	CEFD
Origami 2(DE3)/pET-28b	16	8	8	8	<8	8
Origami 2(DE3)/pMQ28	64	16	64	16	128	128

a PENG, penicillin G; AMP, ampicillin; AMO, amoxicillin; CAR, carbenicillin; CEF, cephalexin; CEFD cefadroxil. The tested concentrations of antibiotics were 8, 16, 32, 64, 128, 256, and 512 μg/ml. The plasmid pMQ28 contains the cloned *macQ* gene, and the plasmid pET-28b is an empty vector control.

Among all AHL acylases previously identified (12–20), MacQ has the broadest substrate specificity toward various AHLs, ranging from C_6_ to C_14_ in length (Table 1). Besides, MacQ was also able to inactivate a variety of β-lactams, including penicillin derivatives (penicillin G, ampicillin, amoxicillin, and carbenicillin) and cephalosporin derivatives (cephalexin and cefadroxil) (Table 2), although known β-lactam acylases mainly degrade only penicillin G, ampicillin, and cephalexin (22, 23). Furthermore, MacQ showed much broader substrate specificity than two AHL acylases (AhlM and *Kc*PGA) known to degrade penicillin G. It has been reported that AhlM was unable to degrade HSLs with a chain smaller than C_8_ and cephalosporin-related β-lactams (19), whereas *Kc*PGA did not show any degradation activity against HSLs with a chain larger than C_8_ (20). Thus, MacQ is a unique bifunctional acylase with broad substrate specificity toward AHLs and β-lactam antibiotics.

### Sequence analysis of MacQ.

**FIG 5:**
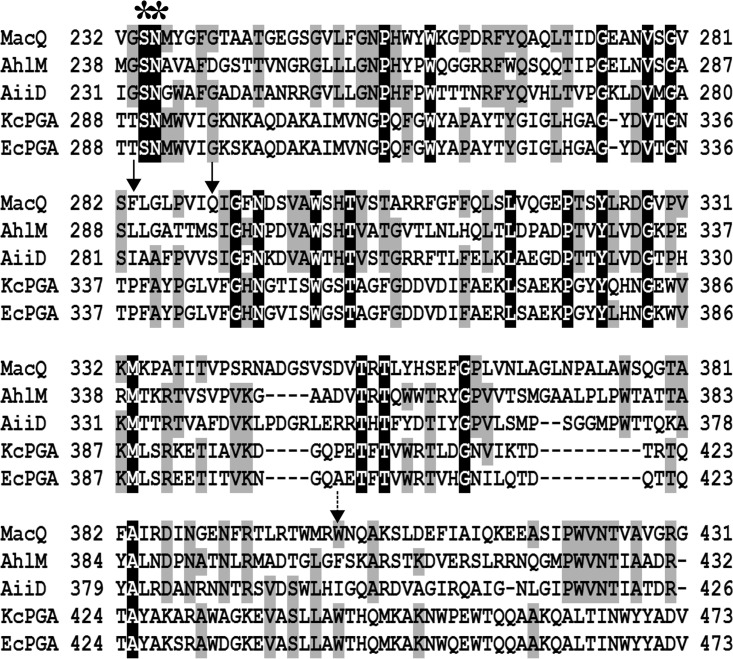
Multiple alignment of amino acid sequences of MacQ and Ntn hydrolase. The black and gray shading indicates identical and similar amino acids, respectively. Conserved residues of relevant to posttranslational modification and catalysis are indicated by asterisks. Highly variant residues known or thought to be involved in substrate specificity are indicated by solid arrows. The amino acid residue related to substrate specificity for penicillin G is indicated by a dotted arrow. MacQ, bifunctional acylase from Acidovorax sp. MR-S7; AhlM, AHL acylase known to degrade penicillin G from Streptomyces sp. M664 (accession no. AY561759); AiiD, AHL acylase from Ralstonia sp. XJ12B (accession no. AAO41113); *Ec*PGA, β-lactam acylase from Escherichia coli ATCC 11105 (accession no. P06875); *Kc*PGA, AHL acylase known to degrade penicillin G from Kluyvera citrophila DSM 2660 (accession no. P07941).

The first 24 residues of MacQ are predicted to be a signal sequence by SignalP (24), implying that MacQ is an extracellular enzyme, as well as the known Ntn hydrolase proteins (19, 25). Indeed, amino acid residues (Ser-234 and Asn-235; Fig. 5) involved in posttranslational modification and catalysis (14, 26, 27) were highly conserved in MacQ and other Ntn hydrolase proteins. This accords with the result of SDS-PAGE analysis of MacQ, implicating that MacQ might be synthesized as a precursor polypeptide and modified into two enzymatically active subunits by posttranslational modification (Fig. 2), as other known acylases (19, 25). Intriguingly, three residues (Ile-282 and Ser-289 in AHL acylase [AiiD] and Trp-443 in *Kc*PGA) associated with the substrate specificity (28, 29) were not fully conserved in MacQ, and we identified alternative residues (i.e., Phe-283, Gln-290, and Trp-401 in MacQ) with the same alignment positions (Fig. 5). We infer that the lack of amino acid sequence conservation may contribute to the broad substrate specificity and bifunctionality of MacQ, although further investigation with crystal structure analysis is required to verify this.

### Phylogenetic analysis of bifunctional acylases.

**FIG 6:**
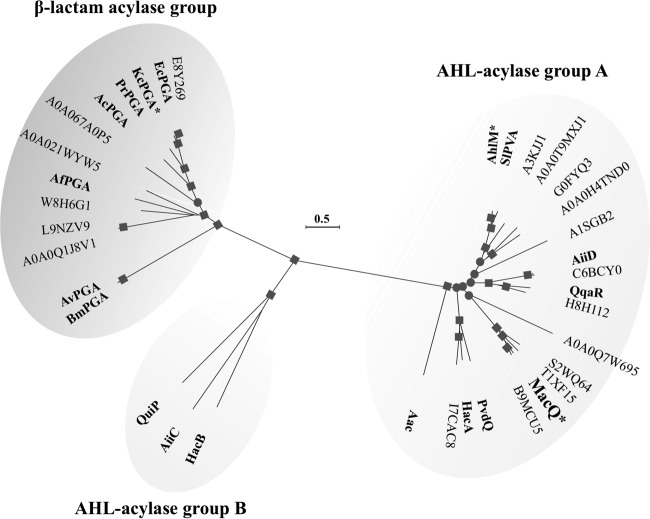
Phylogenetic tree showing relationships between MacQ and Ntn hydrolase proteins. Each of these enzymes was experimentally identified (boldface type), except the proteins designated with UniProt accession numbers. The tree was divided into three groups: β-lactam acylase group (dark gray background) and AHL acylase groups A and B (light gray backgrounds). Amino acids (accession numbers) of AHL acylase groups A and B were as follows: AiiD, from Ralstonia sp. XJ12B (AAO41113); PvdQ, from Pseudomonas aeruginosa PAO1 (AAG05773); AhlM, from Streptomyces sp. M664 (AY561759); QuiP, from Pseudomonas aeruginosa PAO1 (AAG04421); HacA, from Pseudomonas syringae B728a (AAY37014); HacB, from Pseudomonas syringae B728a (AAY39885); AiiC, from Anabaena sp. strain PCC7120 (BAB75623); Aac, from Shewanella sp. strain MIB015 (AB306517); QqaR, from Deinococcus radiodurans R1 (WP_010889514); and SlPVA, from Streptomyces lavendulae ATCC 13664 (AAU09670). Amino acids (accession numbers) of the β-lactam acylase (penicillin G acylase [PGA]) group were as follows: *Ec*PGA, from Escherichia coli ATCC 11105 (P06875); *Kc*PGA, from Kluyvera citrophila DSM 2660 (P07941); *Ac*PGA, from Achromobacter sp. strain CCM 4824 (AAY25991); *Bm*PGA, from Bacillus megaterium ATCC 14945 (Q60136); *Af*PGA, from Alcaligenes faecalis ATCC 19018 (U93881); *Av*PGA, from Arthrobacter viscosus ATCC 15294 (P31956); and *Pr*PGA, from Providencia rettgeri (AAP86197). The bifunctional acylases are indicated by asterisks. Bootstrap values greater than 50% and 90% estimated using neighbor-joining (NJ) and maximum likelihood (ML) methods (1,000 replications) are shown by circle and square symbols at branching points, respectively.

Based on phylogenetic analysis using amino acid sequences, we found that the Ntn hydrolase family proteins were divided into three groups (i.e., one β-lactam acylase group and AHL acylase groups A and B), and that MacQ was classified into AHL acylase group A (Fig. 6). Two AHL acylases, AhlM and *Kc*PGA, known to degrade penicillin G were categorized into AHL acylase group A and a β-lactam acylase group, respectively (Fig. 6). Thus, the bifunctional acylases degrading both AHLs and β-lactams might be broadly distributed among the phylogeny, which implies that the bifunctionality may be conserved in other acylases of a wide variety of microorganisms, perhaps including pathogens. In fact, Ntn hydrolase proteins in this phylogram are derived from phylogenetically diverse bacteria: members of the phyla Actinobacteria, Cyanobacteria, Deinococcus-Thermus, and Proteobacteria, some of which are clinically and agriculturally important pathogens, such as Acinetobacter baumannii ATCC 19606, Mycobacterium tuberculosis ATCC 25618, Pseudomonas aeruginosa PAO1, and Pseudomonas syringae B72a. Further biochemical characterization is necessary for known and putative acylases to verify our assumption.

### Conclusions.

We isolated and characterized a new AHL acylase (MacQ) with the bifunctional capability to degrade not only a variety of AHLs but also multiple β-lactam antibiotics. It has been recognized that antibiotic resistance and quorum quenching are distinct biological functions in bacteria. This study has, however, linked β-lactam degradation with quorum quenching in an explicit way by discovering a bifunctional enzyme. Our findings further provide the possibility that quorum-quenching genes (i.e., AHL acylase genes) would be an alternative source of antibiotic resistance genes.

## MATERIALS AND METHODS

### Bacterial strains, culture media, and growth conditions.

A multidrug-resistant bacterium, Acidovorax sp. strain MR-S7, was previously isolated from activated sludge in a penicillin G production wastewater treatment system (21). Escherichia coli strain DH5α (TaKaRa, Tokyo, Japan) and strain Origami 2(DE3) (Novagen, Madison, WI) were used as the host strains for DNA manipulation and expression of the cloned gene, respectively. E. coli strain MT102 and Pseudomonas putida strain F117 with the *gfp* reporter plasmids pJBA132 and pKR-C12, respectively, were used as biosensors for AHL degradation activity in the bioassay (30, 31). Strain MR-S7, P. putida, and E. coli strains were cultured on Luria-Bertani (LB) agar or in LB broth at 30°C, 30°C, and 37°C, respectively. When appropriate, antibiotics were added at the following concentrations: kanamycin, 50 μg/ml; tetracycline, 20 μg/ml; and gentamicin, 25 μg/ml.

### Cloning and heterologous expression of a putative AHL acylase gene.

To explore the genes encoding AHL acylase or β-lactam acylase, a homology search and protein domain search were conducted using the NCBI BLAST program (http://www.ncbi.nlm.nih.gov/) and the InterProScan sequence search program (http://www.ebi.ac.uk/interpro/search/sequence-search), respectively, against the draft genome sequence data of strain MR-S7 obtained in our previous study (21). Signal sequence prediction analysis was conducted using the SignalP 4.1 server (http://www.cbs.dtu.dk/services/SignalP/). A *macQ* gene coding region was amplified with PrimeSTAR HS DNA polymerase (TaKaRa) using the following primers: For_mac, 5′-GGAATTCCATATGGCCTGCGGAGGCAGCGGC-3′ (NdeI site underlined); and Rev_mac, 5′-CCGCTCGAGCTCCTTCACGGTGGCCGTGCG-3′ (XhoI site underlined). PCR amplification was performed with initial denaturation at 98°C for 5 min, followed by 40 cycles at 98°C for 10 s, and then 68°C for 2.5 min. The PCR product was then gel purified using a QIAquick gel extraction kit (Qiagen, Valencia, CA), digested by each restriction enzyme, and subcloned into expression vector pET-28b (Novagen). The resulting plasmid, pMQ28, was transformed into E. coli strain Origami 2(DE3). To induce recombinant protein overexpression, 0.1 mM isopropyl-β-d-thiogalactopyranoside (IPTG) was added to the E. coli cultures. The cells were harvested by centrifugation at 5,800 × *g* for 10 min after 18 h of cultivation at 18°C, washed with suspension buffer (50 mM Na_2_PO_4_, 300 mM NaCl, 10% glycerol [pH 7.5]), resuspended in the same buffer, and disrupted using an ultrasonic disintegrator (Sonifier 250; Branson Ultrasonics). The cell debris was removed by centrifugation at 5,800 × *g* for 10 min. The supernatant was applied to HIS-Select nickel affinity gel (Sigma-Aldrich, St. Louis, MO), and the His-tagged recombinant protein was purified according to the manufacturer's instructions. The purified protein was treated with marker dye (1% SDS, 1% 2-mercaptoethanol, 10 mM Tris-HCl [pH 6.8], 20% glycerin, and 1 mg/ml bromophenol blue) and heated for 5 min at 98°C. The sample was subjected to a 10% PAGE gel under 30 mA for 80 min. The protein band was stained with Bio-Safe Coomassie brilliant blue (Bio-Rad, Hercules, CA).

### Antibiotic susceptibility testing.

MICs were determined as the lowest concentration of antibiotics preventing visible growth of E. coli strains on the agar. E. coli Origami 2(DE3) harboring plasmid pMQ28 or pET-28b was cultivated in LB broth with kanamycin. After incubation with IPTG, each culture of recombinant E. coli cells was equated and inoculated on LB agar plate with a selected β-lactam antibiotic. All plates were incubated at 37°C for 18 h under ambient atmosphere. The tested antibiotics were penicillin G, ampicillin, amoxicillin, carbenicillin, cephalexin, and cefadroxil, and the final concentrations were 8, 16, 32, 64, 128, 256, and 512 μg/ml.

### Bioassay of AHL inactivation activity.

Ten micrograms of purified MacQ protein was mixed with various AHL solutions at a final concentration of 5 μM, except for OC_6_-HSL (added at 0.115 μM), and dispensed into a well of a 96-well microtiter plate (Becton Dickinson, Franklin Lakes, NJ). The plate was incubated at 30°C for 12 h without agitation. Then, 100 μl of a 5-fold-diluted overnight culture of the reporter strains described above, E. coli strain MT102 and Pseudomonas putida strain F117, was added in equivalent numbers into each well and incubated at 30°C for 4 h without agitation. GFP fluorescence was measured using a SpectraMax Gemini XS microplate spectrofluorometer (Molecular Devices, Sunnyvale, CA) at excitation and emission wavelengths of 485 nm and 538 nm, respectively. The AHLs tested varied in their *N*-acyl side chains, as follows: *N*-hexanoyl-l-homoserine lactone (C_6_-HSL), *N*-octanoyl-l-homoserine lactone (C_8_-HSL), *N*-decanoyl-l-homoserine lactone (C_10_-HSL), *N*-dodecanoyl-l-homoserine lactone (C_12_-HSL), *N*-3-oxo-hexanoyl-l-homoserine lactone (OC_6_-HSL), *N*-3-oxo-octanoyl-l-homoserine lactone (OC_8_-HSL), *N*-3-oxo-decanoyl-l-homoserine lactone (OC_10_-HSL), *N*-3-oxo-dodecanoyl-l-homoserine lactone (OC_12_-HSL), and *N*-3-oxo-tetradecanoyl-l-homoserine lactone (OC_14_-HSL).

### Metabolite analyses of C_10_-HSL and penicillin G degradation.

A purified MacQ protein (100 μg) was mixed with 3 mM C_10_-HSL or 2 mM penicillin G solution and incubated at 30°C for 3 h or 12 h, respectively. After incubation, the digestion mixture was extracted with equal volumes of ethyl acetate three times; thereafter, the combined organic phase was evaporated to dryness in a vacuum. The redissolved samples in methanol were introduced onto a Hitachi M7200A GC/3DQMS system equipped with a DB-5ms capillary column (30 mm by 0.25 mm; J&W Scientific, Folsom, CA) coated with (5%-phenyl)-methylpolysiloxane (250 nm thickness). Helium was used as carrier gas at a flow rate of 1.5 ml/min. The column temperature profile was initially 60°C for 1 min, was increased to 320°C at a rate of 10°C/min, and was finally held at 320°C for 5 min.

### Inhibition of virulence factor production.

In a plant-pathogenic bacterium, Pectobacterium carotovorum subsp. carotovorum, two different AHLs (OC_6_-HSL and OC_8_-HSL) regulate the development of soft rot symptoms on host plants (32, 33). The assay was carried out on potato tubers (each *n* = 5), as described previously (34). Briefly, potatoes were washed with running tap water, sterilized with 70% ethanol, and finally dried under sterile conditions. Escherichia coli expressing MacQ protein was used as a quorum quencher, whereas E. coli harboring an empty vector was used as a nonquenching control. P. carotovorum strain NBRC 3830 was cultivated in growth medium (1% polypeptone, 0.2% yeast extract, 0.1% MgSO_4_ [pH 7.0]) at 30°C for 15 h and mixed with the culture of recombinant E. coli for equal volumes. The reaction mixture was incubated at 30°C for 2 h with gentle shaking and introduced directly into the surface of sliced potato tubers. The tubers were incubated at 28°C under 70% humidity for 2 days. The results were estimated by visual inspection of the infected area.

### Conserved amino acid sequences and phylogenetic analysis.

Multiple-amino-acid-sequence alignment analysis was performed using CLUSTAL W2 and the GENETYX software (Genetyx, Tokyo, Japan). The amino acid sequence of MacQ was aligned with four amino acid sequences of Ntn hydrolase family proteins, including a known β-lactam acylase (*Ec*PGA from Escherichia coli ATCC 11105), AHL acylase (AiiD from Ralstonia sp. strain XJ12B), and two AHL acylases known to degrade penicillin G (AhlM and *Kc*PGA). The phylogenetic trees were constructed with MEGA6 by using the neighbor-joining method based on the JTT matrix-based model (35). Amino acid sequences of experimentally identified Ntn hydrolase proteins were used. In addition, homologous sequences of bifunctional acylases were retrieved from the UniProt database (http://www.uniprot.org/) and clustered using the CD-HIT clustering program (36). Bootstrap resampling analysis using neighbor-joining and maximum likelihood methods (each 1,000 replications) was performed.

### Accession number(s).

The complete nucleotide sequence of the *macQ* gene was deposited in the NCBI GenBank database with accession no. AB702957.
